# Associations among ADHD, Abnormal Eating and Overweight in a non-clinical sample of Asian children

**DOI:** 10.1038/s41598-017-03074-4

**Published:** 2017-06-06

**Authors:** Lian Tong, Huijing Shi, Xiaoru Li

**Affiliations:** 1 Department of Maternal, Child and Adolescent health, School of Public Health, Fudan University/Key Laboratory of Public Health Safety, Chinese Ministry of Education, Shanghai, China; 2 Department of Psychology, School of Social Development and Public Policy, Fudan University, Shanghai, China

## Abstract

Attention-deficit/hyperactivity disorder (ADHD) has been found to be comorbid with obesity in adults, but the association in children is uncertain. Because the underlying mechanism of comorbidity in children has not been researched sufficiently, this study aims to explore the associations among ADHD, abnormal eating, and body mass index (BMI), as well as the mediating effect of depression in children. We conducted a cross-sectional study of 785 primary students in China. The parent-report version of ADHD Rating Scale-IV (ADHDRS-IV), the Child Eating Behaviour Questionnaire (CEBQ) and the Children’s Eating Attitude Test (ChEAT) were used to identify ADHD symptoms and abnormal eating. The Child Behavior Checklist (CBCL) was applied to assess depression. Structural Equation Modeling was carried out to clarify the associations between ADHD symptoms, depression, abnormal eating, and overweight of students. We found that ADHD positively contributed to emotional eating and Bulimia Nervosa symptoms. However, neither emotional eating nor Bulimia Nervosa symptoms was related to BMI in children. We also found that ADHD significantly contributed to depression, and depression directly predicted emotional eating. In conclusion, ADHD increased the risk of abnormal eating in children, while no significant relationship existed between ADHD and BMI. Comorbid depression raised the risk of emotional eating, rather than Bulimia Nervosa symptoms.

## Introduction

Attention-deficit/hyperactivity disorder (ADHD) is a disorder of inattention, impulsivity, and hyperactivity that affects 8% to 12% of children worldwide^1, 2^. It has been suggested that individuals with ADHD may have an elevated risk for obesity, which comes from studies of adults and youth in both clinical and population-based settings^3–6^. However, when looking at a younger age in children, the comorbidity between ADHD and obesity has not been examined sufficiently, especially with the lack of data based on population. A study based on a small sample of clinical data confirmed that ADHD would increase the risk of obesity in boy ADHD patients^7^. Another study based on a national survey with 62887 children and adolescents aged 5 to 17 years suggested that children with ADHD had 1.5 times the odds of being overweight, after adjustment for age, gender, and other demographic information^8^. A recent meta-analysis study revealed that adolescents with ADHD only have 1.1 times of odds of being overweight^9^. In that national survey, a doctor-diagnosed ADHD was used to determine ADHD status, therefore, the finding is based on ADHD patients as well. Few studies have been done in children based on general population.

Furthermore, the potential underlying mechanisms between ADHD, eating disorder, and obesity has not been examined sufficiently^10^. A recent meta-analysis study demonstrated that there are significant overlapping neurobehavioral circuits in ADHD, eating disorder, and obesity in pediatric populations^11^. Research has mainly examined ADHD as a risk factor for obesity^12, 13^. A prospective study indicated that inattention-hyperactivity symptoms at 8 years old were associated with indices of obesity at 16 years old, after adjustment for gender and baseline BMI, rather than the opposite^14^.

With better insight into the potential mechanism of comorbidity, abnormal eating such as Bulimia Nervosa and especially impulsivity-induced eating, is considered to be an important mediator for the association between ADHD and obesity^15^. An updated meta-analysis study indicated that people with ADHD are 3.8 times more likely to present with any eating disorder as a comorbid diagnosis, and the risk increased to 5.7 times for Bulimia Nervosa^16^. Bulimia Nervosa is characterized by recurrent episodes of binge eating, and recurrent inappropriate compensatory behavior in order to prevent weight gain^17^. Community-based studies suggested that Bulimia Nervosa symptoms partially explained the associations between ADHD and obeisty^18, 19^. In addition, emotional eating has been defined as eating in response to a range of negative emotions such as anxiety, depression, and loneliness to cope with negative affect emotional eating^20, 21^. Emotional eating is highly correlated with other eating disorders, like Bulimia Nervosa symptoms, since both of them are related to emotion-focused coping, maladaptive coping strategies, and a strong aversion to negative feelings and stimuli^22–24^. It has been suggested that ADHD symptoms and impulsivity predicted both emotional eating and binge eating in adult women, and such abnormal eating behaviors were, in turn, positively associated with BMI^25^. Similar results have been replicated in males^26^. In addition, adult patients with Bulimia Nervosa and childhood ADHD were more impulsive than patients with Bulimia Nervosa alone, and these patients also displayed more severely disordered eating patterns^27^. ADHD inattention symptoms, depressive symptoms and trait-impulsivity in obese women patients highly predicted their binge eating behaviors^28^.

Considering the sample of children and adolescents, ADHD comorbidity of Bulimia Nervosa symptoms has been discussed in few studies, but the findings are confounding. A study suggested that childhood ADHD diagnosis predicted eating pathology in female adolescents^29^. In another study, both boys and girls with ADHD displayed more Bulimia Nervosa symptoms than controls^30^. Conversely, an inconsistent outcome indicated that no significant differences in rates of Bulimia Nervosa symptoms were identified in children with ADHD when compared to the sex-matched control group^31^. This might be explained by the fact that full Bulimia Nervosa syndrome is not that common in the pediatric group.

It was said that children/adolescents with oppositional defiant disorder (ODD) symptoms showed increased eating in response to external cues, even though binge eating and ADHD symptoms were not associated with disordered eating behaviors in overweight and obese children/adolescents^32^. However, to our knowledge, no study has examined the relationship between ADHD and emotional eating in children.

To date, the evidence regarding the relationships among ADHD, abnormal eating, and overweight is extremely limited in a sample of children. To our best knowledge, only 1 recent study showed that adolescents diagnosed with ADHD showed more binge eating problems, and after adjusting for binge eating, the relationship between ADHD and BMI z-scores was attenuated^33^. The study was carried out based on community mental health clinics, and the effect of binge eating was excluded from the model, so little is known about the mediating effect of abnormal eating on the association between ADHD and obesity. Therefore, the first purpose of this study is to clarify the relationships among ADHD, abnormal eating (including Bulimia Nervosa symptoms and emotional eating), and BMI in children at the population level.

Except for abnormal eating, ADHD is associated with a broader range of psychiatric comorbidity, e.g., depression is a common internalizing disorder^34, 35^. A review of studies in community samples has reported that the rate of depression in youth with ADHD is 5.5 times higher than in youths without ADHD, with rates ranging from 12% to 50%^36^. Children/adolescents with symptoms of depression showed emotional and binge eating^37^. Those with ADHD and comorbid depression had significantly increased risk for substance abuse and suicide compared to children with ADHD or depression alone^35, 37^. Obese women with both of ADHD and eating disorders have higher rates of substance abuse; moreover, inattention symptoms combined with depression predict binge eating^28^. However, little is known about the role of depression between ADHD and eating problems, even though emotional eating and Bulimia Nervosa symptoms are highly correlated with mood. A preliminary study had explored the relationship among them, but the effect of depression was often excluded from the analysis models. For instance, in a clinical sample of severely obese adolescents aged 12 to 17 years, ADHD symptoms were significantly associated with Bulimia Nervosa symptoms after controlling for depressive symptoms^38^. Thus, the third purpose of this study is to identify the mediating effect of depression between ADHD and abnormal eating in children.

## Results

This study mixed a sample with different socioeconomic strata from China. Table 1 showed the statistical difference between the selected three schools in parents’ education level, age and household income. Generally to say, parents in School A have highest socioeconomic level, e.g. high education level, high household income and young parents. On the contrary, the parents in School C have lowest socioeconomic level in the current samples. As displayed in Table 2, boys (mean = 15.3) showed more ADHD symptoms than girls (mean = 11.6; F = 36.33, *P* < 0.0001). The behaviors of emotional undereating in boys (mean = 9.2) was also slightly higher than girls (mean = 8.6; F = 4.54, *P* < 0.05). There were no gender differences in Bulimia Nervosa symptoms, emotional overeating behaviors, and depression. The results indicated that 98 children (12.9%) were considered obese and 150 children (19.7%) were considered overweight in the sample. There were more boys than girls in both the overweight and obesity classifications (χ^2^ = 13.449, *P* < 0.01). Children in obesity/overweight group have a slightly higher ADHD score (mean = 14.3) than children in normal weight group (mean = 13.0; t = −1.85, *P* = 0.064). Children in obesity/overweight group have more Bulimia Nervosa symptoms (mean = 1.3) than normal weight group (mean = 1.1), but the difference is not statistically significant. Similarly, there are no significant differences in emotional overeating/undereating and depression symptoms within two groups.

**Table 1:** The variance in the demographic information of participated families of three elementary schools.

	School A n (%)	School B n (%)	School C n (%)	χ^2^_MH_
Father’s education level				
Illiteracy/Elementary school	6(1.8)	9(3.4)	13(7.2)	102.2****
Middle school	98(30.1)	82(31.4)	106(59.2)	
Senior school	71(21.8)	87(33.3)	47(26.3)	
Undergraduate college	135(41.5)	78(29.9)	13(7.3)	
Graduated college	15(4.6)	5(1.9)	0(0)	
Mother’s education level				
Illiteracy/Elementary school	17(5.3)	21(7.9)	53(29.8)	146.6*****
Middle school	94(29.4)	86(32.5)	93(52.3)	
Senior school	80(25.0)	78(29.4)	25(14.0)	
Undergraduate college	127(39.7)	77(29.1)	7(3.9)	
Graduated college	2(0.6)	3(1.1)	0(0)	
Annual household income (US$)				
≤3000	9(2.9)	16(6.3)	25(15.1)	71.3****
3000–6000	30(9.7)	34(13.4)	22(13.2)	
6000–9000	52(16.9)	51(20.2)	39(23.5)	
9000–12000	38(12.3)	29(11.5)	38(22.9)	
12000–15000	63(20.5)	38(15.0)	26(15.7)	
≥15000	116(37.7)	85(33.6)	16(9.6)	
Father’s age				
<30	5(1.6)	2(0.7)	8(4.5)	46.4****
30–40	256(80.5)	154(58.8)	112(62.9)	
40–50	54(16.9)	97(37.0)	54(30.3)	
50–60	3(1.0)	9(3.5)	4(2.2)	
Mother’s age				
<30	6(1.9)	6(2.3)	13(7.4)	43.5****
30–40	288(90.6)	210(79.5)	120(67.8)	
40–50	23(7.2)	46(17.4)	42(23.7)	
50–60	1(0.3)	2(0.8)	2(1.1)	
Single child				
Yes	239(73.5)	160(59.3)	55(30.4)	
No	86(26.5)	110(40.7)	126(69.6)	
ADHD symptoms				1.0
Yes	247(90.8)	294(89.4)	162(88.0)	
No	25(9.2)	35(10.6)	22(12.0)	
Total	329(41.9)	272(34.6)	184(23.4)	

****p < 0.0001.

**Table 2:** The partial correlation among variables controlling for gender.

	Total	Boys	Girls	F
	mean (SD)	mean (SD)	mean (SD)	
ADHD^a^	13.4 (8.9)	15.3 (9.5)	11.6 (7.9)	36.33****
Bulimia Nervosa symptoms^b^	1.2 (2.4)	1.4 (2.5)	1.2 (2.2)	2.34
Emotional overeating^c^	6.1 (2.4)	6.2 (2.5)	6.0 (2.3)	1.34
Emotional undereating^c^	8.9 (3.7)	9.2 (3.6)	8.6 (3.7)	4.54*
Depression^d^	3.1 (3.3)	3.1 (3.4)	3.0 (3.1)	0.34
BMI	**n (%)**	**n (%)**	**n (%)**	**χ** ^**2**^
Obesity	98 (12.9)	63 (8.3)	35 (4.6)	
Overweight	150 (19.7)	90 (11.8)	60 (7.9)	13.449**
Normal or thinness	513 (56.4)	245 (32.2)	268 (35.2)	

^a^ADHD Rating Scale-IV; ^b^Children’s Eating Attitude Test; ^c^Child Eating Behaviour Questionnaire; ^d^Child Behavior Checklist. * *P* < 0.05, ** *P* < 0.01, ****P < 0.0001.

The results in Table 3 indicated the correlations between ADHD, abnormal eating, and depression after adjustment for gender. We found that ADHD was positively correlated with Bulimia Nervosa symptoms (r = 0.18916, *P* < 0.0001), emotional overeating (r = 0.31435, *P* < 0.0001), emotional undereating (r = 0.27953, *P* < 0.0001), and depression (r = 0.48574, *P* < 0.0001). Bulimia Nervosa symptoms was positively correlated with emotional overeating as well (r = 0.15883, *P* < 0.0001). Emotional undereating was positively correlated with depression (r = 0.31332, *P* < 0.0001). Emotional overeating is highly correlated with emotional undereating as well (r = 0.47550, *P* < 0.0001). No significant correlation was found between BMI and ADHD or abnormal eating.

**Table 3:** The partial correlation among variables controlling for gender.

	ADHD	Bulimia Nervosa symptoms	Emotional overeating	Emotional undereating	Depression
Bulimia Nervosa symptoms	0.18916****				
Emotional overeating	0.31435****	0.15883****			
Emotional undereating	0.27953****	0.06490	0.47550****		
Depression	0.48574****	0.06122	0.27988****	0.31332****	
BMI	0.00667	0.04496	−0.00340	−0.03110	−0.04129

****P < 0.0001.

**Figure 1:**
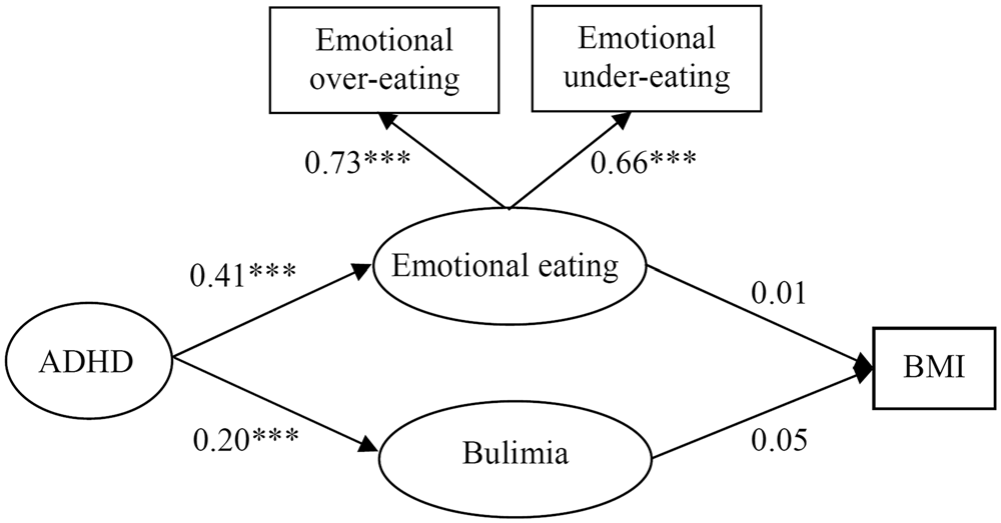
SEM model 1 testing correlations among ADHD symptoms, emotional eating, bulimia and BMI in children. *|t| < 1.96, **|t| < 2.58, ***|t| < 3.28.

In Fig. 1, SEM model 1 explored the effect pathway among ADHD, emotional eating and BMI. We found that ADHD positively contributed to emotional eating (*β* = 0.41, *P* < 0.001) and Bulimia Nervosa symptoms (*β* = 0.20, *P* < 0.001) in children. However, neither emotional eating (*β* = 0.01, *P* > 0.05) nor Bulimia Nervosa symptoms (*β* = 0.05, *P* > 0.05) was related to BMI. The proposed Model 1 represents an excellent fit to the data (GFI = 0.994). The details are provided in Table 4. The loading factors for the 2 variables of emotional eating were 0.73 to 0.66.

**Table 4:** Comparing model 1 with model 2 in model fitness indices.

	Chi-Square	P value of Chi-Square	Goodness of Fit Index (GFI)	Adjusted GFI (AGFI)	RMSEA Estimate	RMSEA Lower 90% Confidence Limit	RMSEA Upper 90% Confidence Limit
Model 1	10.3997	<0.05	0.994	0.9703	0.0598	0.0229	0.1013
Model 2	70.6114	<0.0001	0.963	0.8896	0.1177	0.0937	0.1434

**Figure 2:**
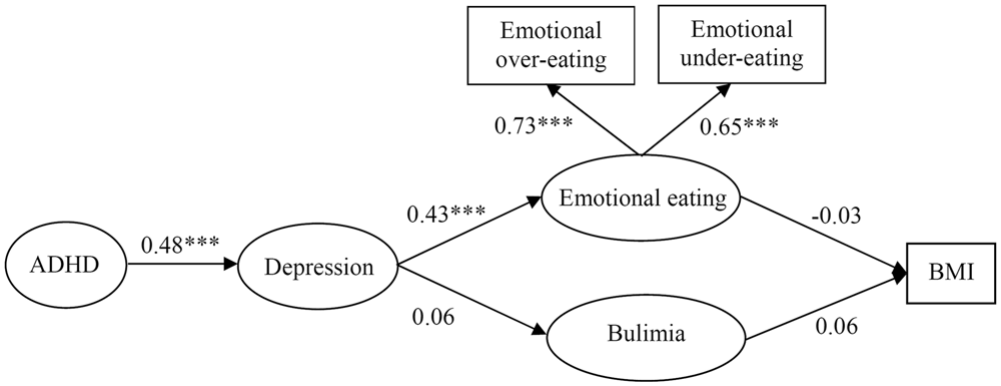
SEM model 2 testing correlations among ADHD symptoms, depression, emotional eating, bulimia and BMI in children. *|t| < 1.96, **|t| < 2.58, ***|t| < 3.28.

In Fig. 2, compared with SEM model 1, the mediating effect of depression was added between ADHD and abnormal eating in SEM model 2. We found that depression would directly predict emotional eating (*β* = 0.43, *P* < 0.001), rather than bulimia (*β* = 0.06, *P* > 0.05). ADHD significantly contributed to depression (*β* = 0.48, *P* < 0.001). It suggested that ADHD not only contributed to abnormal eating directly, but also had an effect on emotional eating through depression when two symptoms were comorbid. Similar to model 1, neither emotional eating (*β* = 0.03, *P* > 0.05) nor Bulimia Nervosa symptoms (*β* = 0.06, *P* > 0.05) was related to BMI. SEM model 2 also demonstrated a good fit to the data (GFI = 0.963). The loading factors for the 2 variables of emotional eating were 0.73 to 0.65.

## Discussion

Even though an eating disorder has been demonstrated to be an important mediating factor between ADHD and obesity in adults, the evidence is very limited in children^15^. The current study indicated that ADHD symptoms in children contributed to their Bulimia Nervosa symptoms. Impulsivity might be the cause of ADHD comorbidity with Bulimia Nervosa symptoms, since impulsivity is considered to be a central symptom for both disorders^30^. Impulsive symptoms such as interrupting, intruding, and difficulty waiting one’s turn are part of the diagnostic criteria for the Combined Type of ADHD, which has been conceptualized as a problem of inhibition and impulse control. Impulsive personality traits are thought to be central to Bulimia Nervosa, and up to 40% of individuals with Bulimia Nervosa show multi-impulsivity symptoms in other life domains beyond eating, such as risky sexual behavior, stealing, and drug abuse^39^. Even though a study with a small sample of college students indicate that impulsivity hasn’t mediated the relationship between ADHD and binge eating disorder^40^, impulsivity has been shown to predict the onset of Bulimia Nervosa symptoms 9 months later, among adolescent girls in a community sample^41^. Therefore, impulsivity and lack of inhibition in children with ADHD may play a role in triggering Bulimia Nervosa^42^. Additionally, some genic feature might be the cause of the relationship. It had been suggested that the dopaminergic genes, especially the dopamine receptor D4 (DRD4), have been associated independently with both binge eating and ADHD^43^. Reward Deficiency Syndrome caused by abnormalities of dopaminergic genes could be a transdiagnostic feature between ADHD, eating disorder, and obesity^44, 45^.

In addition, a unique finding in the present study was that ADHD can predict emotional eating behaviors in children, including both emotional overeating and undereating. Some people eat less in the face of strong emotions, but some may turn to impulsive or binge eating to struggle with emotional distress. To our best knowledge, only 1 study examined the relationship between ADHD and emotional overeating, but no specific interpretation was discussed. A nationwide twin study suggests that children with ADHD show significant higher prevalence of restrictive eating behaviors than their counterparts, especially for girls^46^. A speculated mechanism is that the stress and negative emotion faced by children with ADHD increased the risk of emotional eating behaviors, because individuals engage in emotional eating only when they are experiencing negative emotions^47^. Emotional eating is associated with a range of emotional problems, such as low self-esteem, social anxiety, and feelings of inadequacy^48^. Children with ADHD often suffer from low social recognition, poor school achievement, and high accompaniment with mood disorders, which may contribute to emotional eating^49^. Meanwhile, those children probably perceive subjectively greater stress that may result in anxiolytic quality of eating^50^.

Given emotional eating is closely related with emotional problems, ADHD is highly comorbid with emotional disorders, especially depression^51^. Our present study clarified the mediating effect of depression between ADHD and eating behaviors. As expected, we found that depression mediated the relationship between ADHD and emotional eating in children, rather than ADHD and bulimia. Depression has long been documented as a significant comorbid condition of eating disorders among female adolescents^52^. It has been suggested that the presence of depressive symptoms and ADHD inattention symptoms can predict the present of binge eating in obese women^28^. Depressive symptoms would predict eating disorders in 13 to 18-year-old girls over a 4-year period, but not the reverse^53^. One finding in our study indicated that children with ADHD who are comorbid with depression would have an increased risk of emotional eating. To summarize shortly, the complex relationships between ADHD and eating disorder not just rely on single factors (e.g., impulsivity, genetic abnormalities, depression), but the interactions among these factors might interpret the associations. It has been suggested that not only ADHD neuropsychological functioning could alter information processing for food-related decision making, but also changes in nutritional or emotional state promoted by eating disorder could make ADHD individuals more prone to disinhibition^16^.

Taking BMI into consideration, although the relationships between ADHD and eating disorders have been confirmed in this study, no significant relationship among ADHD, eating disorder and BMI was established in children. This finding is consistent with a previous study in which the diagnosis of ADHD was not associated with any of the obesity trajectories (no obesity, childhood obesity, adolescent obesity, and chronic obesity) in the general pediatric population (age ranged from 9 to 16 years)^54^. To date, only a few studies based on ADHD patients demonstrated that ADHD could increase the risk of obesity in children^7, 8^ and none of these were carried out at the population level. The close relationship between ADHD and eating disorders in children potentially increase the risk of obesity when they are grown. Obesity has been found to be a long-term consequence of binge eating disorder in epidemiological studies^55^. Another interpretation is that the median age for the onset of an eating disorder is between 12 and 13 years of age^56^; this means children who are comorbid with ADHD and eating disorders might take a long time to become obese.

## Limitations

The current study is a cross-section design, so a longitudinal study is needed to explore the causality among ADHD, abnormal eating, and BMI, even though the method of SEM is suggested to be a good method to explore the causal relationship for cross-sectional data. ADHD was assessed with the parent-reported questionnaire, rather than an interview by a professional. In addition, except for abnormal eating and depression, multi-factors related to both ADHD and BMI should be taken into consideration to construct a SEM model, such as sleep problems and screen time.

## Method

### Participants

This study was approved by the medical ethics committee of Fudan University (Shanghai, China). The corresponding author confirms that this study was performed in accordance with the approved social experiment guidelines and regulations. Informed consent was obtained from legal parents of all participating students. A total of 785 primary students, aged 9 to 13 years (Mean = 10.6, SD = 1.1), and their parents were recruited by stratified random sampling (from third grade to fifth grade) of 3 primary schools in Shanghai, China. Among the sample, 409 (52.1%) were boys (mean age = 10.6, SD = 1.1) and 376 (47.9%) were girls (mean age = 10.6, SD = 1.1).

## Measures

### ADHD

Both the student self-reported questionnaire and parent-filled questionnaire were used in this study. The 2 questionnaires were matched by student identification (ID). The specific assessment instruments covered in the 2 questionnaires were reported as follows:

The parent-report version of the ADHD Rating Scale-IV (ADHDRS-IV) was used to assess ADHD symptoms^57^. The ADHDRS-IV is an 18-item ADHD assessment scale, and it consisted of 2 subscales, inattention and hyperactivity-impulsivity, each containing 9 items. Each item was mapped onto 1 of the 18 Diagnostic and Statistical Manual of Mental Disorders, 4^th^ Edition (DSM-IV) symptoms of ADHD. Parents were required to rate the frequency of each of the ADHD symptoms, which occurred over the previous 6 months, on a 5-point Likert scale that ranged from 0 to 4 (never [0], rarely [1], sometimes [2], often [3], and very often [4]). The sum of all the scores on the18 items resulted in a total score that ranged from 0 to 54. The reliability and validity of the home version of ADHDRS-IV had been verified in a sample of Chinese children aged 6 to 17 years^58^. Cronbach’s alpha was 0.92 for the ADHDRS-IV in this sample.

#### Abnormal eating

Two separate scales were used to assess abnormal eating behaviors:

1. Emotional eating
  The Child Eating Behaviour Questionnaire (CEBQ) is a parent-report questionnaire designed to assess variation in eating styles among children^59^. It has been shown to have a robust factor structure and good internal reliability^60^. Two subscales involving emotional overeating and emotional undereating were used in our present study. Parents were asked to rate their child’s eating behavior on a 5-point Likert scale that ranged from 1 to 5 (never [1], rarely [2], sometimes [3], often [4], and always [5]). Sample scale items included, for example, “My child eats less when s/he is angry; my child eats more when s/he is happy; my child eats more when worried; and my child eats more when annoyed.” Cronbach’s alpha was 0.86 for both subscales of emotional overeating and emotional undereating.
2. Bulimia Nervosa symptoms

The Children’s Eating Attitude Test (ChEAT), a modified version of the Eating Attitudes Test (EAT) is a self-reported questionnaire filled out by children themselves^61^. The psychometric properties and the application of ChEAT in different cultures have been certified in previous studies^62–65^. The ChEAT contained 26 items which were rated on a 6-point Likert scale that ranged from 0 to 3: always (3), very often (2), often (1), sometimes (0), rarely (0), and never (0). A subscale of Bulimia Nervosa was also used in this study. Sample scale items included, for example, “I have gone on eating binges where I feel that I might not be able to stop” and “I give too much time and thought to food.” The Cronbach’s alpha for the bulimia nervosa subscale was 0.67.

#### Depression

The parent of each participant completed the Chinese version of the Child Behavior Checklist for ages 4 to 18 years (CBCL/4–18). The CBCL is a very common tool because its psychometric properties and Chinese version have been certified in previous studies^66, 67^. The subscale of depression contained 17 items. Sample scale items included, for example, “Deliberately harms self or attempts suicide; Cries a lot; and Worries.” Each item was rated on a 3-point Likert scale that ranged from 0 to 2 (not true [0], somewhat or sometimes true [1], and very true or often true [2]). The Cronbach’s alpha for the depression subscale was 0.75.

#### Body Mass Index

Body Mass Index (BMI) was calculated from height and weight (weight [kg]/height^2^ [m^2^]) measured with the participant wearing indoor clothing and standing in stocking feet. The height and weight were measured by a healthcare provider in the school hospital. Specific age and gender BMI cut-off points were used to define overweight and obesity according to the International Obesity Task Force (IOTF) Body Mass Index Cut-Offs^68^.

### Statistical analysis

The data was analyzed by Statistical Analysis System (SAS) 9.3 (Institute Inc., Cary, NC, USA). Partial correlation analysis was used to examine the correlation between ADHD, abnormal eating, and depression. The relationship between ADHD symptoms, emotional eating, Bulimia Nervosa symptoms, and BMI was analyzed by using Structural Equation Modeling (SEM).
